# Sleep quality, anxiety, and depression among perioperative caregivers of children with tethered cord syndrome: a cross-sectional survey analysis

**DOI:** 10.3389/fped.2025.1580053

**Published:** 2025-04-23

**Authors:** Jinrun Lin, Hao Li, Suqin Xia, Qing Cheng, Dou Lin, Dehong Fan, Qing Zhuo, Yulan Kang

**Affiliations:** Fujian Children’s Hospital (Fujian Branch of Shanghai Children’s Medical Center), College of Clinical Medicine for Obstetrics & Gynecology and Pediatrics, Fujian Medical University, Fuzhou, China

**Keywords:** tethered cord syndrome, children, caregivers, sleep quality, anxiety, depression

## Abstract

**Objectives:**

This study aimed to investigate the relationship between sleep quality, anxiety, and depression among perioperative caregivers of children with tethered cord syndrome.

**Methods:**

A cross-sectional study design was employed to recruit primary caregivers of children undergoing surgery for tethered cord syndrome from Fujian Children's Hospital between February 2022 and December 2023. Participants were required to complete some questionnaires, including the Pittsburgh Sleep Quality Index (PSQI), the Self-Rating Anxiety Scale, and the Self-Rating Depression Scale. Statistical analyses like chi-square tests, t-tests, and logistic regression were carried out to evaluate the associations between sleep quality, anxiety, and depression.

**Results:**

A total of 149 caregivers were included in the study and completed all the questionnaires. The findings indicated that the prevalence of sleep disorders among caregivers was 31.5%. Correlation analysis revealed a significant positive association between anxiety and depression. Multifactorial regression analysis identified younger age of the child, presence of comorbidities, extended duration in a prone position postoperatively, and prolonged caregiving time as independently significant factors contributing to sleep disorders in caregivers.

**Conclusions:**

The study determined the occurrence of sleep disorders among primary caregivers of children with tethered cord syndrome. Furthermore, where the young age of the child, the presence of comorbidities, the time the child spent in a prone position after the operation, and the time spent caring for the child were significant factors. Future studies should explore the potential mechanisms and causal connections between caregiver sleep disorders and these crucial factors.

## 1 Introduction

Tethered Cord Syndrome (TCS) is caused by various congenital and acquired reasons that cause tension on the conus medullaris. This can lead to a descent in the position of the conus or thickening of the terminal filum, which restricts spinal cord movement. This manifests as progressive neurological dysfunction, causing a series of and developmental abnormalities, such as lower limb dysfunctions, defecation, and urination disorders (1, 2). Congenital TCS can occur at any age, but is most common in newborns and children (3), with an incidence rate of 0.05‰–0.25‰ in newborns (4).

Surgical intervention is the only effective treatment method and should be carried out promptly after diagnosis (5). In the management of childrenwith Tethered Cord Syndrome (TCS), the perioperative phase is crucial, and nursing staff play a vital role. Before the operation, the presence of a mass in the lumbosacral region negatively affects the child's rest, nutrition, and daily activities. Large masses with thin membranes are prone to rupture, while pilonidal sinuses, due to their deep location and hair coverage, are difficult to detect and tend to get infected. Neurological complications like incontinence may lead to incontinence-associated dermatitis if not managed properly (6). Restrictive positioning causes the child's discomfort and presents many challenges for caregivers. After the operation, complications are common, often showing as impaired wound healing, cerebrospinal fluid leakage, subcutaneous effusion, intracranial infection, and neurological problems such as neurogenic bladder and rectum, as well as sensory-motor disorders and deformities in the lower extremities. In severe cases, these complications can develop into intraspinal and central infections, possibly resulting in life-threatening situations (7).

Due to the limited self-care ability of children, the perioperative daily care and rehabilitation for children with TCS are mainly taken care of by caregivers, usually the child's parents and educators. These primary caregivers are crucial in supporting and encouraging the child to gain confidence in overcoming the disease. They take the main responsibility of caregiving and are indispensable for the child's recovery. During the child's illness, caregivers encounter significant psychological, emotional, and social challenges as they devote a lot of time and energy to providing care. The young age and self-care limitations of children with TCS, as well as the recurrent nature of the condition, impose considerable psychological stress and a heavy burden on their immediate family caregivers (8).

The psychological well-being and quality of life of nursing personnel during the perioperative period are very important for the recovery ofchildren with TCS. Empirical evidence reveals that the sleep quality, anxiety, and depression of nursing staff can significantly affect their caregiving efficacy, which in turn influences the child's recovery. A comprehensive review suggests that when caregivers stay overnight in the hospital withchildren, psychological and environmental factors have a considerable impact on the caregivers' sleep quality. Hence, it is necessary to explore the relationship between sleep quality, anxiety, and depression among caregivers of children with Tethered Cord Syndrome (TCS) during the perioperative period to enhance care quality and facilitate the child's recovery. In recent years, the psychological status and quality of life of nursing staff have received increasing academic attention. Nevertheless, there is a considerable shortage of research specifically concentrating on the sleep quality, anxiety, and depression of nursing staff during the perioperative period of children with spinal cord tethering, and the existing studies mainly focus on the stress, burden, and coping mechanisms of caregivers.

This cross-sectional study intended to investigate the sleep quality, anxiety, and depression of caregivers of children with TCS during the perioperative period, and the interconnections between these elements, with the aim of enhancing the sleep quality of caregivers and promoting the perioperative recovery of children.

## 2 Materials and methods

### 2.1 Study design

The sleep quality of primary caregivers of children with spinal cord embolism during the perioperative period was rigorously assessed using a questionnaire survey administered at the Children's Hospital of Fujian Province, China. This study received approval from the Institutional Ethics Committee of Fujian Provincial Children's Hospital, and informed consent was obtained from all participants.

### 2.2 Study population

The study consecutively enlisted the primary caretakers of children undergoing perioperative therapy for tethered cord syndrome at Fujian Children's Hospital from February 2022 to December 2023. The sample magnitude was ascertained in accordance with the actual quantity of children with tethered cord syndrome who underwent surgery in the neurosurgery division.

### 2.3 Instruments

In this study, a nurse was assigned to distribute and collect data from caregivers using three well-established and reliable scales, namely the Pittsburgh Sleep Quality Index (PSQI), the Zung Self-Rating Anxiety Scale (SAS), and the Zung Self-Rating Depression Scale (SDS).

#### 2.3.1 Pittsburgh sleep quality Index (PSQI)

The PSQI is a self-administered questionnaire comprising 19 items designed to evaluate caregivers' sleep quality (9). The questionnaire assesses seven components: subjective sleep quality, sleep latency, total sleep duration, habitual sleep efficiency, sleep disturbances, use of sleep medication, and daytime dysfunction. Each component is rated on a 4-point Likert scale ranging from 0 to 3. A cumulative score of 5 or above indicates the presence of a sleep disorder.

#### 2.3.2 Zung self-assessment anxiety and depression scales (SAS and SDS)

The SAS was used to evaluate caregiver anxiety, and the SDS to assess caregiver depression (10). Both of these scales comprise 20 parameters, and each parameter is rated from 1 to 4. The scores of each parameter are added together and then multiplied by 1.25 to get the final score.

### 2.4 Data gathering

Before the survey, caregivers were informed consent. The researcher explained the questionnaire to the participants with standard instructions. For those who could read, they were told how to complete the questionnaire on their own and what was needed for it. For those who couldn't read, the researcher read the questions and answers in a neutral tone so that the caregiver could make a choice, and helped with the completion process. The questionnaires were collected right away, their completeness was checked, and any omissions or errors were quickly fixed. Altogether, 149 valid questionnaires were collected.

### 2.5 Statistical analysis

Statistical analyses were performed using SPSS version 29.0 (IBM Corp., Armonk, NY). Descriptive statistics like frequencies, percentages, means, and standard deviations (SD) were employed to evaluate the demographic characteristics of the sample including children and their primary caregivers, and also to assess the sleep quality, anxiety, and depression levels of the primary caregivers. Univariate and multivariate regression analyses were carried out to determine significant predictors of sleep quality among primary caregivers. Variables with a *p* -value less than 0.05 in the univariate analysis were regarded for inclusion in the multivariate analysis. Odds ratios (OR) and 95% confidence intervals (CI) were computed.

## 3 Results

### 3.1 Study participants

A total of 152 questionnaires were distributed to the caregivers, and 149 valid responses were received, resulting in a response rate of 98%.

### 3.2 Demographics

Table 1 showed the basic characteristics of the children. The average age of the children was 4.05 ± 3.65 years, with a relatively broad age range. More than half of them (55.70%) were the only child in their family. A considerable number of the children (74.50%) had complications associated with tethered cord syndrome. Moreover, the average time they spent in bed was 13.89 ± 5.95 h, suggesting a considerable amount of bed rest.

**Table 1:** Demographic characteristics of 149 children.

Variables	No. (%) or mean ± SD
Age, years	4.05 ± 3.65
Gender	
Male	86 (57.72%)
Female	63 (42.28%)
only-child status	83 (55.70%)
Presence of complications	111 (74.50%)
Average time in prone position, hours	13.89 ± 5.95

Table 2 presents the characteristics of 149 caregivers. The average age was 35.53 ± 8.40. Most are female (69.80%). The majority were parents (93.96%). The average number of hours spent caring for children per day was 15.19 ± 6.32.

**Table 2:** Demographics of 149 caregivers.

Variables	No. (%) or Mean ± SD
Age, years	35.53 ± 8.40
Gender	
Male	45 (30.20%)
Female	104 (69.80%)
Relationship with the child	
Parents of the child	140 (93.96%)
Grandparents of the child	9 (6.04%)
Income	
≤5,000	23 (15.4%)
5,000–10,000	56 (37.6%)
10,000–20,000	51 (34.2%)
≥20,000	19 (12.8%)
Occupation	
Worker	5 (3.4%)
Corporate employee	45 (30.2%)
Professional	36 (24.2%)
Housewife	29 (19.5%)
Freelancer	14 (9.4%)
Service staff	4 (2.7%)
Other	16 (10.7%)
Duration of care, hours	15.19 ± 6.32

Table 3 titled “PSQI, SAS, and SDS Scores” presents the results. The total mean score of the Pittsburgh Sleep Quality Index (PSQI) scale was 5.60 ± 2.99. Among its components, “Time to Fall Asleep” had the highest mean score of 1.28 ± 0.75, and “Use of Sleep Medication” had the lowest mean score of 0.07 ± 0.26. Additionally, the mean scores for the Self-Rating Anxiety Scale (SAS) and the Self-Rating Depression Scale (SDS) were 42.27 ± 6.92 and 43.33 ± 6.25 respectively.

**Table 3:** Presents the distribution of sleep quality, anxiety, and depression among the caregivers studied.

Items	Mean ± SD
Subjective sleep quality	0.60 ± 0.73
Sleep latency	1.28 ± 0.75
Sleep duration	1.17 ± 0.90
Sleep efficiency	0.55 ± 0.92
Sleep disturbances	0.94 ± 0.35
Sleep medication usage	0.07 ± 0.26
Daytime dysfunction	1.01 ± 1.04
Total of PSQI	5.60 ± 2.99
Total of SAS	42.27 ± 6.92
Total of SDS	43.33 ± 6.25

Table 4 illustrates the correlation between the Pittsburgh Sleep Quality Index (PSQI), the Self-Rating Anxiety Scale (SAS), and the Self-Rating Depression Scale (SDS). Notably, a significant positive correlation was observed between the SAS and SDS; however, no correlation was identified between the PSQI and either the SAS or SDS.

**Table 4:** Correlation analysis of PSQI, SAS, and SDS.

Variables	1	2	3	4	5	6	7	8	9
1. PSQI	1								
2. Sleep duration	0.370**	1							
3. Sleep quality	0.581**	0.370**	1						
4. Use of sleep medication	0.107	0.062	0.050	1					
5. Daytime functioning	0.729**	0.290**	0.305**	−0.052	1				
6. Time to fall asleep	0.467**	0.172*	0.092	0.033	0.249**	1			
7. Sleep efficiency	0.696**	0.470**	0.231**	−0.030	0.415**	0.102	1		
8. Sleep disturbances	0.418**	0.203**	0.140	0.048	0.296**	0.165**	0.186**	1	
9. SAS	−0.002	−0.059	−0.202*	0.121	0.112	0.262**	−0.183**	0.063	1
10. SDS	−0.011	−0.144	−0.090	0.163*	0.034	0.159	−0.102	0.164*	0.341**

** *p* < 0.01.

* *p* < 0.05.

Table 5 presented the results of the univariate regression analyses, demonstrating several crucial factors influencing the sleep quality of caregivers. Four of these factors were significant predictors in the multivariate model. Caregivers looking after younger children were more prone to have sleep disorders, with an adjusted odds ratio (aOR) of 1.832 (95% CI: 1.038–1.304, *p* = 0.009). For instance, when a caregiver was taking care of a younger child, the risk of developing a sleep disorder was elevated. Likewise, the existence of comorbidities in the child makes the caregiver more vulnerable to a sleep disorder (aOR = 3.439, 95% CI: 1.141–5.717, *p* = 0.023). A longer duration of the child being in a postoperative prone position also heightened the probability of caregivers having sleep disorders (aOR = 1.116, 95% CI: 1.044–1.195, *p* < 0.001). Furthermore, caregivers who had been caring for the child for an extended period were also more inclined to have a sleep disorder (aOR = 1.099, 95% CI: 1.033–1.170, *p* = 0.003).

**Table 5:** Offered a thorough regression analysis concerning the predictors of caregiver sleep disturbances.

Variables	Poor sleep quality PSQI ≥ 7 *N* = 47	Good sleep quality PSQI < 7 *N* = 102	Univariable OR (95% CI)	*p*-value	Multivariable OR (95% CI)	*p*-value
Characteristics of children						
Age, years	2.87 ± 2.86	4.59 ± 3.86	1.163 (1.038–1.304)	0.009	0.832 (0.728–0.950)	0.007
Sex, female	18	45	1.272 (0.628–2.577)	0.504		
Only-child status	28	55	1.259 (0.625–2.538)	0.519		
Presence of complications	38	63	2.614 (1.141–5.989)	0.023	3.439 (1.335–8.860)	0.011
Average time in prone position, hours	16.55 ± 6.50	12.66 ± 5.27	0.891	<0.001	1.116 (1.044–1.195)	0.001
Characteristics of caregivers						
Age, years	34.72 ± 9.25	35.90 ± 8.01	1.018 (0.975–1.063)	0.426		
Sex, female	34	70	0.836 (0.392–1.795)	0.647		
Parents of the child	44	96	0.917 (0.219–3.835)	0.905		
Duration of care, hours	17.85 + 6.74	13.97 ± 5.75	0.906 (0.865–.0959)	<0.001	1.099 (1.033–1.170)	0.003
Income			5.334	0.502		
≤5,000	7	16				
5,000–10,000	22	34				
10,000–20,000	13	38				
≥20,000	5	14				
Occupation			2.673	0.445		
Worker	2	3				
Corporate employee	12	33				
Professional	13	23				
Housewife	13	16				
Freelancer	3	11				
Service staff	1	3				
Other	3	13				
SAS	42.21 ± 6.58	42.29 ± 7.10	1.002 (0.953–1.053)	0.945		
SDS	43.09 ± 6.01	43.44 ± 6.39	1.009 (0.955–1.067)	0.744		

## 4 Discussion

Sleep is an essential physiological requirement for the body, vital for upholding normal bodily and mental operations (11). Studies indicate that sleep disruptions are prevalent among caregivers of hospitalised children due to diverse elements, inevitably influencing the caregiver's psychological well-being and subsequently the child's recuperation procedure (12). This research aimed to assess the sleep quality, anxiety, and depression magnitudes of caregivers managing spinal embolism syndrome. It is crucial for nurses to forestall, recognise, and resolve caregiver sleep issues to enhance satisfaction with hospitalisation, caregiver productivity, and the child's postoperative convalescence.

In this study, the prevalence of sleep disorders among caregivers of children with tethered spinal cord syndrome was found to be 31.5%, along with varying degrees of anxiety and depression. This result was similar to the 39% prevalence of sleep disorders among caregivers of peopleundergoing hemodialysis and peritoneal dialysis reported in a previous study (13). That study also showed that caregivers with poor sleep quality had higher levels of anxiety and depression, and depressive symptoms were identified as a factor affecting sleep quality. However, in the present study, there was no significant correlation between depressive symptoms, anxiety symptoms, and sleep quality, but caregivers still had obvious sleep disorders, anxiety, and depressive symptoms. These results might be due to differences in sample size, study design, or measurement tools. When looking at the sleep quality of caregivers, their mental health status must not be ignored. The anxiety and depression symptoms of caregivers may require psychological interventions and social support to relieve these conditions, thereby improving their overall quality of life and caregiving ability (14, 15).

The study found that a child's young age is a crucial factor causing caregivers' sleep disorders. Younger children need more physical care, like diaper changes and feeding, which raises the caregivers' physical burden and makes them wake up multiple times at night to look after the child. Consequently, this influences the caregivers’ sleep continuity and quality (16). Additionally, the caregivers of younger children may receive less social support since their social activities might be restricted by caregiving responsibilities. The absence of social support can enhance the pressure on caregivers and affect their sleep (17).

Furthermore, the presence of complications in the child is a significant factor inducing sleep disorders among caregivers. For example, complications associated with tethered spinal cord syndrome render the care more intricate. It necessitates additional medication, regular medical examinations, or specialized care processes. All these signify that caregivers need to be vigilant at night. Moreover, these complications can give rise to higher medical costs, escalating the family's financial load. This can bring about psychological strain for the caregiver and have a harmful impact on their sleep quality (18).

The study also identified the extended duration spent in the prone position post-surgery as a notable contributor to caregivers' sleep disturbances. Due to the surgical incision, children are required to maintain a prone position, which may demand greater physical effort from caregivers and necessitate more frequent nighttime monitoring. This requirement can lead to multiple awakenings to assess the child's condition and prevent further complications (19).

Moreover, the duration of caregiving has been identified as a significant determinant of sleep disorders among caregivers. As the time devoted to caring for a child increases, caregivers are likely to experience more frequent sleep disruptions. The chronic stress associated with prolonged caregiving responsibilities can adversely affect caregivers' sleep architecture, hindering their ability to achieve deep sleep or maintain uninterrupted sleep cycles. Interruptions such as nighttime awakenings to attend to the child or the inability to rest during the day due to caregiving duties cumulatively contribute to the risk of developing sleep disorders (20).

Providing professional nursing guidance, optimizing care plans, and reducing the caregivers' burden through appropriately arranging care times and offering professional caregiving training are essential (21). Offering nighttime nursing support can alleviate the burden on caregivers, such as the assistance of nurses or care staff as needed. Educating caregivers about the importance of the prone position after surgery and how to provide effective care without disturbing their own sleep is also crucial.

This study had several limitations. The subjects were recruited from a single hospital, and the main variables were evaluated with subjective scales as reported by the subjects. Moreover, the sample size was limited by the number of children undergoing surgery for tethered spinal cord syndrome. Future research should involve multiple hospitals, increase the sample size, incorporate objective measures of sleep quality, and investigate the correlation between the sleep quality of caregivers and that of the children. Further studies should also explore various intervention measures, like adjusting the times of being in the prone position and using assistive devices, to lessen the impact on caregivers' sleep.

## 5 Conclusion

The study discovered that the prevalence of sleep disorders among caregivers was 31.54%, and these disorders were associated with various degrees of anxiety and depressive symptoms. The crucial factors influencing sleep include the child's age, the average time spent in the prone position, the presence of comorbidities before treatment, and the average time spent caring for the child. For future research, it is extremely significant to further investigate the underlying mechanisms and causal connections between sleep disorders and related factors.

## Data Availability

The datasets presented in this study can be found in online repositories. The names of the repository/repositories and accession number(s) can be found in the article/Supplementary Material.
